# Organic field-effect optical waveguides

**DOI:** 10.1038/s41467-018-07269-9

**Published:** 2018-11-15

**Authors:** Guangyao Zhao, Huanli Dong, Qing Liao, Jun Jiang, Yi Luo, Hongbing Fu, Wenping Hu

**Affiliations:** 1 Beijing National Laboratory for Molecular Sciences, Key Laboratory of Organic Solids, Institute of Chemistry, Chinese Academy of Sciences, Beijing, 100190 China; 2 School of Chemistry and Chemical Engineering, University of the Chinese Academy of Sciences, Beijing, 100039 China; 3 Beijing Key Laboratory for Optical Materials and Photonic Devices, Department of Chemistry, Capital Normal University, Beijing, 100037 China; 4 Heifei National Laboratory for Physical Sciences at the Microscale, University of Science and Technology of China, 230026 Hefei, China; 5 Tianjin Key Laboratory of Molecular Optoelectronic Sciences, Department of Chemistry, School of Science, Tianjin University & Collaborative Innovation Center of Chemical Science and Engineering, Tianjin, 300072 China

## Abstract

Integrating electronics and photonics is critically important for the realization of high-density and high-speed optoelectronic circuits. However, it remains challenging to achieve this target due to the difficulty of merging many different areas of science and technology. Here, we show an organic integrated optoelectronic device, namely, organic field-effect optical waveguide, integrating field-effect transistor and optical waveguide together. In such device, the propagation of optical waveguide in the active organic semiconductor can be tuned by the third terminal—the gate electrode of transistor, giving a controllable modulation depth as high as 70% and 50% in parallel and perpendicular directions of charge transport versus optical waveguide, respectively. Also, the optical waveguide with different directions can turn the field-effect of the device with the photodependence ratio up to 14800. The successful integration of active field-effect transistor with semiconductor waveguide modulator expands opportunities for creating scalable integration of electronics and photonics in a chip.

## Abstract (web summary)

Despite recent advances in organic optoelectronics development, integration of electronics and photonics in a chip remains a challenge. Here, the authors demonstrate organic field-effect optical waveguides that control propagating photons by the electric field produced in an organic transistor.

## Introduction

Optoelectronic integration has become indispensable as the essential requirements of modern life for high-density connection of electronic and photonic devices.^1–10^ For example, the discovery of the field-effect transistor,^2^ wherein the field effect is created and influenced by the gate voltage, controlling the “conducting channel” and current of the transistor, founded today’s computer science, microelectronics, and information technologies. However, the integration of photonic devices as well as electronic and photonic elements is full of challenges since the weak interaction between photons.^3,11–13^ For organic optoelectronic devices, fortunately, Hide et al.^4^ suggested an application of semiconducting polymers as solid-state laser materials. Sirringhaus et al.^14^ invented an all-polymer semiconductor integrated device with a high-mobility conjugated polymer field-effect transistor driving a polymer light-emitting diode of similar size. Ho et al.^15^ demonstrated composites of nanoparticles and conjugated polymers that exhibit composition-tunable optical constants for use in semiconducting photonic structures. Hepp et al.^16^ reported a light-emitting field-effect transistor based on a tetracene thin film, which integrated a light-emitting diode and field-effect transistors into individual devices. These achievements have greatly contributed to the development of organic optoelectronics;^17–19^ however, optoelectronic integration of electronics and photonics in a chip remains challenging in this field due to the difficulty of merging many different areas of science and technology.

Photons can be imagined as a self-propagating transverse oscillating wave of electric and magnetic fields according to Maxwell’s equations (Supplementary Fig. 1a). Hence, during the propagation of photons in optical waveguides, it has the potential to be tuned by the external electric field.^10,20–25^ It is also well-acknowledged that a stationary charge produces an electric field, a charge moving at constant speed produces electric and magnetic fields, and a charge that is accelerated will produce variable electric and magnetic fields. Hence, taking a field-effect transistor into consideration, charges transport in its conducting channel from source to drain electrodes, and such charge transport process will be potentially influenced by the incident optical waves according to electromagnetic wave theory,^20^ which on the other hand will also modulate propagation of an optical signal in the active medium (Supplementary Fig. 1b). Moreover, as one of the critical devices of electronic logic, the three-terminal transistors that can be controlled by the gate voltage possess signal amplification and switching characteristics, which are seen as the promising device architecture toward high-density and high-speed on-chip integrated optoelectronic circuits. Several types of conceptual optical transistors have been proposed for photonic circuits, namely, quantum well optoelectronic switching devices, field-effect plasmonic modulator, etc.^26–28^ But integration of the active electronic transistors with semiconductor waveguide modulators has rarely been exploited for the realization of direct electro-optic modulators with simultaneously controlling both the light and charge that carries propagation.^29,30^

Herein, we show an organic optoelectronic integrated device, namely, an organic field-effect optical waveguide (OFEW) where the propagating photons can be tuned by the electric current produced in the organic field-effect transistor (OFET), and vice versa. Such extraordinarily convenient voltage control of waveguide behavior can be elucidated by the change and mismatch of energy alignment between molecules due to the formation of charged molecules when electric current flows in device operation. Experimental results demonstrate that the modulation depth for the propagation of an optical waveguide can reach as high as 70% and 50% when photon transport is parallel and perpendicular to the conducting channel, respectively. While under the laser light illumination (with powers of 500 nW to 5 μW), the modulated highest photodependence ratio of such a field-effect transistor can be up to 14800. The successful demonstration of such OFEW opens an avenue and concept to construct active high-density and high-speed optoelectronic integrated circuits on a chip for optical information and communication applications.

## Results

### Preparation and characterization of CHICZ single crystals

**Fig. 1:**
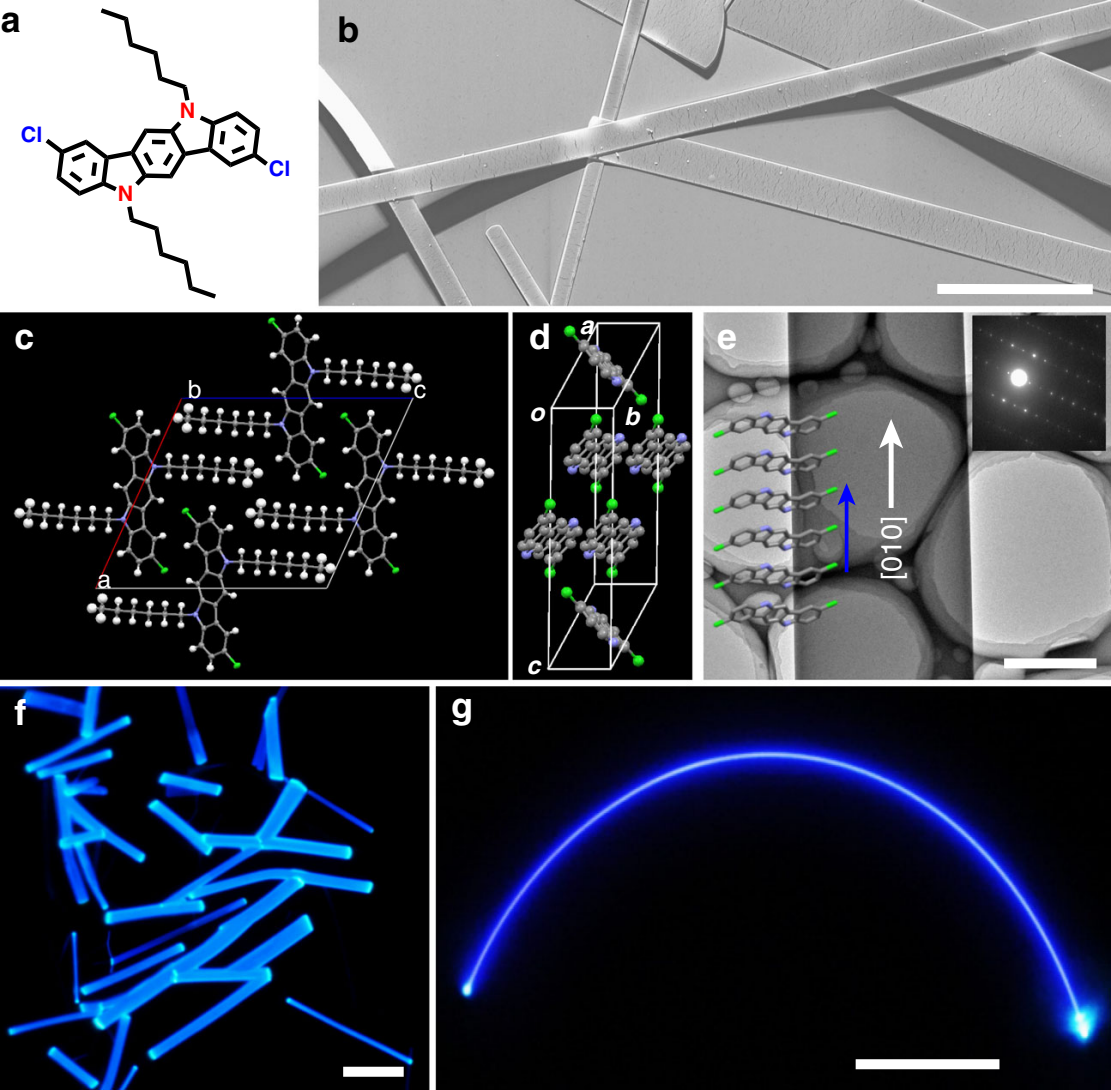
Characterizations of CHICZ single crystals. **a** Molecular structure of CHICZ. **b** SEM image of CHICZ crystal ribbons. Scale bar: 20 μm. **c** and **d** Molecular packing in CHICZ single crystal seen from different directions. **e** TEM image and its corresponding SAED pattern of an individual CHICZ single crystal in which CHICZ molecules are packing along the b axis with π−π stacking. Scale bar: 1 μm. **f** and **g** Fluorescence microcopy images of CHICZ ribbons showing less loss coefficients in long distance and good flexibility. Scale bar: 50 μm

Compared with inorganic semiconductors, organic semiconductors, as a new generation of semiconducting materials, take the advantages,^12,17–19,31^ including tailoring functions by molecular design, ideal flexibility, solution processibility, low in cost, as well as possessing the capacity for creating highly complex integrated optoelectronic systems on planar substrates. To demonstrate the concept of our OFEWs, here, an organic semiconductor, 2,8-dichloro-5,11-dihexyl-indolo(3,2-b)carbazole (CHICZ, Fig. 1a), is used as a model system that can be extended to more complicated fully conjugated systems. The choice of CHICZ is because that it is a pentacene analog with efficient charge transport property. But different from pentacene without fluorescence, CHICZ simultaneously shows strong solid-state fluorescence (Supplementary Fig. 2) as well as excellent stability due to a large energy gap of ∼2.79 eV.^32^

As promising building blocks for high-performance optoelectronic devices, organic micro- and nanocrystals play key roles on efficient charge transport and stable polaritons with long-range propagation due to their high crystallinity, no grain boundary, and minimum defects.^19,33,34^ To carry out our investigation, CHICZ single crystals are first grown by physical vapor transfer (PVT) system^35^ with argon (Ar) carrier gas by carefully controlling the material evaporation condition (Supplementary Fig. 3). As shown in Fig. 1b, regular ribbon-like crystals can be obtained, with a length of tens to hundred micrometers and width of several to tens of micrometers (e.g., a ribbon crystal with a length of over 300 μm and a large two-dimensional size within a thin-film crystal with a width of over 200 μm, as shown in Supplementary Fig. 4). Atomic force microscope (AFM) images demonstrate that these crystals normally have regular rectangular sections with a height of hundreds of nanometers (Supplementary Figs. 5–6). The surface of the crystals is very flat and atomically smooth, further indicating the high quality of obtained CHICZ crystals, which is crucial for reducing the light-scattering loss and obtaining efficient light waveguide propagation in crystals.^36,37^

Single-crystal data of CHICZ demonstrate that it belongs to a *P2*_*1*_*/n* monoclinic space group with unit cell parameters of *a* = 16.759(5) Å, *b* = 4.4914(14) Å, *c* = 18.515(7) Å, and *β* = 114.283(5)^o^.^32^ In crystals, CHICZ molecules adopt a face-to-face slipped *pi*-stacking motif with an intermolecular distance of 3.45 Å for π-π stacking along the *b* axis (Fig. 1c, d). The pitch and roll angles are 10.4^o^ and 26.0^o^, respectively, suggesting efficient orbital overlap between the adjacent molecules and effective charge transport along this direction. X-ray diffraction patterns (XRD) of CHICZ single crystals could be indexed according to its single-crystal data. Obviously, only the multistage (h0l) (h = –l) diffraction patterns are observed (Supplementary Fig. 7), indicating the preferable molecular orientation of CHICZ crystals with (h0l) crystalline planes parallel to the substrate. Transmission electron microscopy (TEM) image and its corresponding selected area electron diffraction (SAED) patterns are shown in Fig. 1e. The same pattern is observed at different parts along the ribbon, indicating that the whole ribbon is a single crystal. The SAED pattern could be indexed with its single crystal lattice constants, and it confirms that the crystal grows along the [010] direction, i.e., the π–π stacking direction of CHICZ molecules. Such direction of this molecular packing is beneficial for charge transport.

Interestingly, CHICZ crystals also show a strong blue emission. Bright light spots are observed at the ends and edges of crystals (Fig. 1f and Supplementary Fig. 5c–i), indicating that they are excellent candidates as optical waveguides. More attractively, ribbons with a length of over 300 μm (Fig. 1g) not only show a strong blue emission and ideal waveguide features, but also excellent flexibility, indicating the potential application of the crystals in flexible optoelectronic devices. In order to accurately evaluate the propagation loss property of our CHICZ crystals, measurements based on the spatially resolved photoluminescence (PL) spectra of an individual ribbon are performed with a 408-nm focused laser beam (which is accurately shifting the excitation laser spots at one end and collecting the PL signal from another end, Supplementary Fig. 8). Two directions, parallel and perpendicular to the crystal long axis, are carried out in our experiment. According to equation (1), optical loss coefficient (*α*) of guided light in the fundamental modes is obtained^38^

1 $$a = - 10 \times \log \left( {I_{\mathrm{out}}/I_{\mathrm{in}}} \right) \times {{L}}^{ - 1}$$

where *I*_in_ and *I*_out_ are the intensities of incident and out-coupled lights, and *L* is the propagation distance. The *α* values for CHICZ crystals at 455 nm along and perpendicular to the ribbon are determined to be 10 and 20 dB mm^−1^, respectively. These values are quite low among organic semiconductors,^39,40^ confirming the excellent optical waveguide features of CHICZ crystals.

To investigate the electronic performance of the obtained CHICZ ribbon crystals, top-contact, bottom-gate OFETs of an individual CHICZ crystal are fabricated based on octadecyltrichlorosilane (OTS)-modified Si/SiO_2_ (300 nm) substrates using an isolated technique^41^ (Supplementary Fig. 9). The devices exhibit typical OFET properties (Supplementary Fig. 10), and the field-effect mobility and the on/off ratio are calculated to be 0.52 cm^2 ^V^−1^ s^−1^ and 2×10^6^, respectively. Moreover, the corresponding threshold voltage of the device is around zero voltage. These excellent field-effect properties together with the excellent optical waveguide properties in CHICZ crystals (Fig. 1) indicate their potential application in our proposed integrated optoelectronic device, OFEWs.

### Device fabrication and optoelectronic modulation of OFEWs

**Fig. 2:**
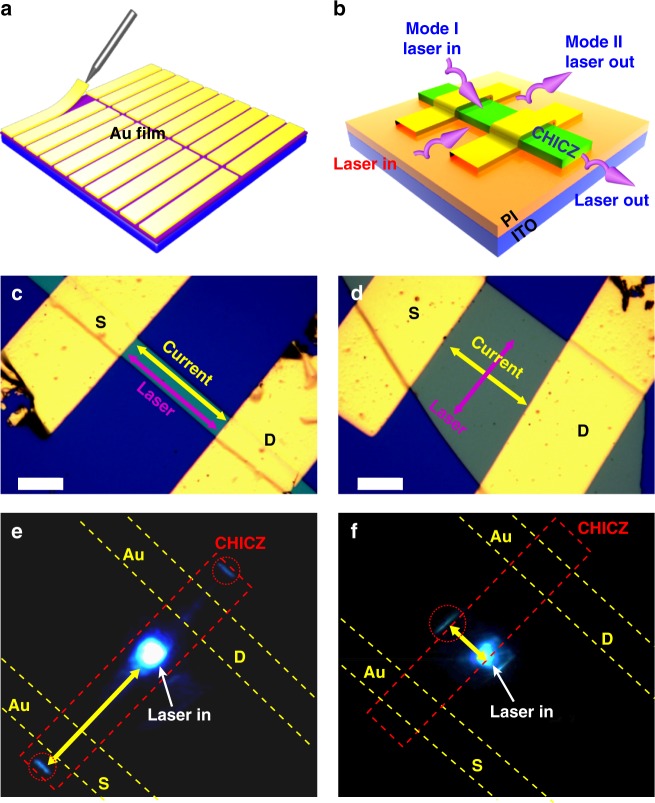
Schematic and actual device of CHICZ-based OFEWs. **a** Gold stripes prepared for source and drain electrodes by “gold stripes stick technique”. **b** Schematic of an OFEW constructed based on an individual CHICZ crystal ribbon with gold stripes as source and drain electrodes. Two models for optical waveguide direction, along the conducting channel (model I), and perpendicular to the conducting channel (model II). **c** Device working in model I, current transport is parallel with the optical waveguide direction. Scale bar: 20 μm. **d** Device working in model II, current transport is perpendicular to the laser, where a large thin crystal is selected for easy experimental operation. Scale bar: 20 μm. **e** and **f** Laser in and out of the devices working in modes I and II, respectively, demonstrating a typical optical waveguide feature in the active device

To construct a stable OFEW device for the following measurement, several points should be considered in the experiment. First, transparent substrates are necessary because the laser illumination and waveguide intensity measurement are from two sides of the device. So here, indium tin oxide (ITO) and polyimide (PI) are selected instead of the former Si and SiO_2_ in OFETs as the gate electrode and insulator layer. Second, to ensure the easy detection of waveguide performance, the ends of CHICZ ribbon crystals should not be covered completely by the electrodes in the device. The specific device fabrication of CHICZ-based OFEW is depicted in Figs. 2a, b. First, Au thin film is pre-deposited on a Si wafer by thermal evaporation. Then, a small piece of Au film, approximately 30 μm × 150 μm, is cut and peeled off from the Si substrate by the tip of the mechanical probe and transferred onto the CHICZ ribbon as a mask for source or drain electrodes deposition (Fig. 2a). After that, the mask gold strip is peeled off and the final device of the individual CHICZ ribbon crystal is obtained, as shown in Fig. 2b (a prototype device is also shown in Supplementary Fig. 11). The incident laser beam is guided into a CHICZ crystal through two ways, i.e., parallel and perpendicular to the conducting channel of the transistors (Fig. 2c–f), to investigate the effect of electro–photo coupling (the measurement system, including OFETs, optical waveguides and laser systems, etc., is shown in Supplementary Fig. 12).

**Fig. 3:**
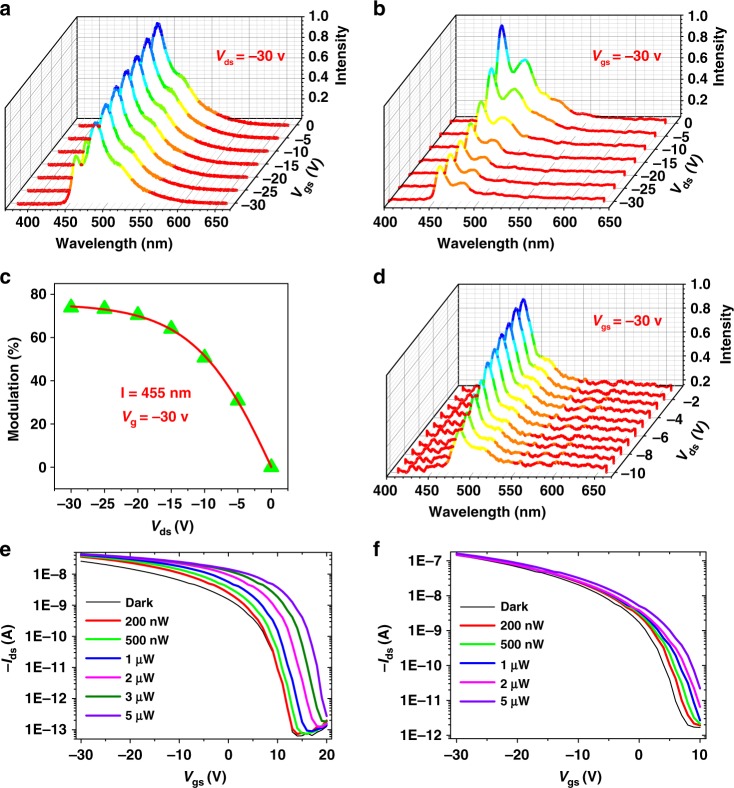
Modulation features of CHICZ-based OFEWs. **a**–**d** Field-effect modulation on an optical waveguide of CHICZ ribbons. **a**–**c** Working in mode I. **a** PL intensity dependence on gate voltage. **b** PL intensity dependence on source–drain voltage. **c** Modulation percentage of the waveguide intensity tuned by different source–drain voltages. **d** PL intensity dependence on source–drain voltage working in mode II. **e** and **f** Optical waveguide modulation on field-effect performance. Transfer characteristic dependence on a different laser illumination working in **e**, mode I and **f**, mode II, respectively

The modulation of field effect on optical waveguides is shown in Fig. 3. At the parallel mode I, that is, the incident laser is parallel to the conducting channel of the transistor (Figs. 2c, e), the output intensities of the waveguide exhibit a strong dependence on the gate bias. Here, modulation-degree *M* is used to characterize the field effect on waveguides. It is calculated by the equation *M* = (1–(*I*_on_*/I*_off_))×100%, where *I*_on_ and *I*_off_ are the intensities with and without voltage bias. At *V*_ds_ = –30 V, *M* is over 25% during V_g_ shifting from 0 to −30 V) (Fig. 3a), confirming the obvious field effect on a waveguide in CHICZ crystals. More interestingly, at a constant electric field, e.g., *V*_g_ = –30 V, the field effect on the waveguide is more significant, as shown in Fig. 3b, and the *M* value is over 75% during *V*_ds_ shifting from 0 to −30 V (Fig. 3c), indicating that the interaction between photons and charges is more notable during the current increase. A similar phenomenon is also observed in a perpendicular mode II, but the incident laser is perpendicular to the conducting channel of the transistor (Figs. 2d, f). The dependence of PL intensity on source–drain voltage is more significant than that on gate voltage, but the modulation degree *M* is over 50% (Fig. 3d). The results clearly demonstrate that our designed OFEW device is approachable by using CHICZ single crystal and the field effect on a waveguide is more significant when the channel current is increasing. In comparison, it is found that without the gate voltage applied and under the same source–drain voltage of –30 V, the maximum *M* value obtained in the experiment is only 10% (Supplementary Fig. 13), suggesting the importance of a larger lateral electric field induced by the field effect on the resulting increased *M* values, that is, a signal amplification effect. It should also be noted that in our experiments, the intensity modulation is accomplished without changing the dominant peak position at *λ* = 455 and 480 nm, and the source/drain voltage is unloaded until the PL intensity is stable to avoid optical cleaning for organic materials (Supplementary Fig. 14).

The modulation of an optical waveguide on transistor performance is also examined. Under different laser intensities, the corresponding transfer characteristics are collected with a constant *V*_ds_ = –30 V, and *V*_gs_ scanning from 20 to −30 V. The results are shown in Figs. 3e and f. *I*_ds_ increases with laser intensity demonstrating a nice photodependence, and the devices work like phototransistors.^42^ Here, a photodependence ratio, which is defined as the (*I*_laser_*–I*_dark_)/*I*_dark_ (where *I*_laser_ and *I*_dark_ is the *I*_ds_ current under laser illumination and without laser illumination at the same *V*_gs_ and *V*_ds_), is used to characterize the effect of laser illumination on field-effect performance. In parallel mode I, with the waveguide propagation direction along the electric current, the photodependence ratio is significantly increased, up to 14800 under 5 μW laser illumination(Fig. 3e). However, in perpendicular mode II, i.e., with the waveguide propagation direction perpendicular to the conducting channel, a very low photodependence ratio (only ~100) is obtained under the same (5 μW) laser illumination (Fig. 3f).

### Theoretical mechanism simulation of OFEWs

**Fig. 4:**
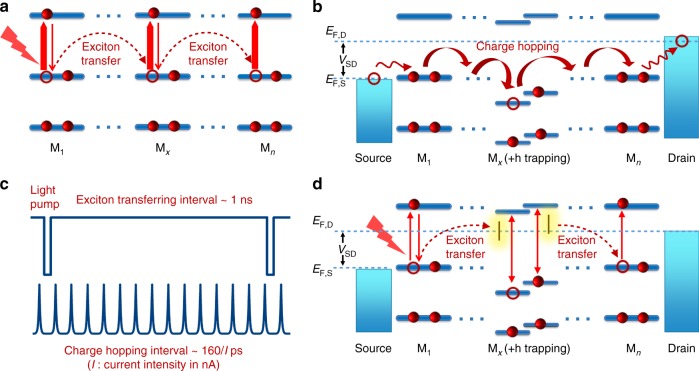
The underlying mechanism of voltage control of waveguide behavior. **a** The exciton transferring in the molecular orbital energy scheme of an aggregated polymer system (M_1_, …, M_*x*_, …, M_*n*_) under photo excitation and no external voltage. **b** The (hole) charge hopping process driven by external voltage in the polymer without photoexcitation. **c** The time scale of normal exciton transfer and charge hop processes. **d** The resonant-energy transferring of photo generated excitons is suppressed by the mismatch of energy gap in M_*x*_ induced by hole trapping due to external voltage control

The underlying mechanism of such extraordinarily convenient voltage control of waveguide behavior can be elucidated in Fig. 4. On one hand, for excitons (electron/hole pairs) generated by light illumination in CHICZ without an external electric field, the resonant-energy transferring process dominates the migration of excitons. As shown in Fig. 4a, the energy schemes based on the molecular orbitals show clearly that the CHICZ molecules (M_1_, the _x_, and the _n_) meet the prerequisite of an identical energy gap for efficient exciton transfer. On the other hand, for the system under an external (source–drain) voltage modulation without photoexcitation (Fig. 4b), the charge hop process becomes dominant. When a hole ( + h) is hopping through M_1_ to M_*n*_ in our system, there are chances that the hole might be trapped in a molecule M_*x*_, which instantly changes the energy scheme/molecular orbitals. One can look into the timescale for the above two processes in Fig. 4c. Normally, the transferring of an exciton takes about 1 ns. The charge hopping rate depends on the electric current, so we can estimate a time interval of 160/I ps for a current intensity of *I* through the organic polymer system, which is always far shorter than the exciton transferring time. Therefore, for our system under both photoexcitation and external voltage in Fig. 4d, the transferring of photogenerated excitons could encounter a large number of hopping charges as long as the hopping current *l* is strong enough. In this case, a hole ( + h) is trapped by a molecule M_*x*_, whose energy scheme will be greatly changed and will not match with other molecules any more. Under such situation, the resonant-energy transferring process and the consequent waveguide will be heavily suppressed by the hole trapping, through which we could achieve efficient voltage control of a waveguide. Such working process is further confirmed by the deep theoretical calculations, as shown in Supplementary Fig. 15. The results clearly demonstrate that no matter for the single molecule and dimers, if a charge (positive or negative) is trapped by molecules when electric currents are driven by gate or source–drain voltage, the frontier orbitals would be effectively up- or downshifted, which reduces the highest occupied molecular orbital (HOMO)–the lowest unoccupied molecular orbital (LUMO) gap significantly (i.e., 1.1–1.7 eV and 3.0–3.1 eV reduced for a single molecule and dimers, respectively). These break the energy match between adjacent molecules and thereby shut down the channel for resonant-energy transferring, leading to the suppression on the output optical waveguide properties.

## Discussion

In conclusion, an innovation of concept for organic integrated optoelectronic devices, organic optoelectronic integrated devices, OFEWs, is proposed and approached by using CHICZ single crystals. The devices demonstrate a successful control of photon propagation by an electric field. When the guide wave is parallel to the conducting channel of the transistor, waveguide intensity modulation is up to 70%, when the guide wave is perpendicular to the conducting channel of the transistor, the modulation is also up to 50%. The field-effect performances of OFEWs are also tunable by optical waveguides with an anisotropic property. With the propagation direction of the optical waveguide along the conducting channel, the photodependence ratio is up to 14800 under 5 µW of laser illumination, while the photodependence ratio is only about 100 with a waveguide perpendicular to the conducting channel under the same laser illumination. Considering the quanta features of photons and electrons, probably, our OFEW is a quantum device, wherein the photon propagation is closely related with the behavior of charges.

## Methods

### Materials

CHICZ is synthesized in our laboratory.^32^ Polyimide (PI) is synthesized, as described elsewhere,^43^ and used as an insulator material.

### Device fabrication

Two kinds of substrates, ITO/PI and Si/SiO_2_, are used. ITO glasses are cut into 2-cm × 2-cm pieces, then cleaned by pure water, pure acetone, and finally blew by pure nitrogen gas. After that, a 12% solid content precursor polyamic acid (PAA) solution in N,N-dimethyl-acetamide is spin-coated on ITO at a speed of 2000 rpm for 30 s, then heated at 140 ^o^C for 1 h to remove the solvent, and subsequently heated to 300 ^o^C to produce a PI thin film, which is used as an insulator layer. CHICZ single crystals are grown by the PVT method and are transferred onto PI/ITO substrates as a semiconducting and waveguide material. In total, 40–50-nm-thick gold source and drain electrodes are deposited on top of CHICZ single crystals through thermal evaporation with a shadow mask of gold layer. In order to observe the fluorescence signal modulation on the end of a CHICZ single crystal, one terminal of a single crystal should not be covered by the gold electrode. In total, 300-nm SiO_2_ on n^++^-doped Si wafer is used as a substrate. Before device fabrication, substrates are cleaned with pure water, pure acetone, piranha solution (H_2_SO_4_:H_2_O_2_ = 7:3), pure water, pure ethanol, and blew by pure nitrogen gas, which are then modified with a self-assembled monolayer of octadecyltrichlorosilane (OTS). The device fabrication is the same as that on the PI/ITO substrate.

### Characterizations of CHICZ single crystals

The morphology and crystallinity of the CHICZ single crystals are characterized by the scanning electron microscopy (SEM, Hitachi S-4800), Olympus BX51, AFM (Digital Instrument, Nanoscopy IIIa), XRD (Rigaku D/max 2500 with Cu K source Å), and TEM (JEOL 1011 operated at 100 kV), respectively. Bright-field optical images and PL images are taken from an inverted fluorescence microscope (OLYMPUS FV1000-IX81), by exciting the samples with the UV band (330–380 nm) of a mercury lamp.

### Characterizations of OFETs and OFEWs

All OFET characteristics are measured using a Keithley 4200-SCS System under an ambient environment at room temperature. The CHICZ single-crystal OFEW devices are locally excited with a focused 408-nm Ar ion laser (spot size ~2 μm). The fluorescence spectra are measured with fluorescent spectroscopy liquid-nitrogen cooled CCD (SPEC-10–400B/LbN, Roper Scientific). Fluorescence images are recorded using an Olympus research inverted system microscope (FV1000-IX81, Tokyo, Japan) equipped with a charge-coupled device (CCD, Olympus DP71, Tokyo, Japan) camera.

## Electronic supplementary material

Supplementary information

## Data Availability

The authors declare that the data supporting the findings of this study are available from the corresponding author upon reasonable request.

## References

[1] Wada O (1994). Optoelectronic integration: physics, technology and applications. South Afr. J. Occup. Ther..

[2] Sedra, A. S. & Smith, K. C. Microelectronic Circuits. 5th edn, (Oxford, New York, 2004).

[3] Ramo S, Whinnery J, Van Duzer T (1984). Fields and waves in communications electronics.

[4] Hide F (1996). Semiconducting polymers: a new class of solid-state laser. Mater. Sci..

[5] Duan X, Huang Y, Agarwal R, Lieber CM (2003). Single-nanowire electrically driven lasers. Nature.

[6] Xu Q, Schmidt B, Pradhan S, Lipson M (2005). Micrometre-scale silicon electro-optic modulator. Nature.

[7] High AA, Novitskaya EE, Butov LV, Hanson M, Gossard AC (2008). Control of exciton fluxes in an excitonic integrated circuit. Science.

[8] Krasavin AV, Zayats AV (2012). Photonic signal processing on electronic scales: electro-optical field-effect nanoplasmonic modulator. Phys. Rev. Lett..

[9] Davoyan A, Engheta N (2014). Electrically controlled one-way photon flow in plasmonic nanostructures. Nat. Commun..

[10] Cui QH (2018). Asymmetric photon transport in organic semiconductor nanowires through electrically controlled exciton diffusion. Sci. Adv..

[11] Davanco M (2017). Heterogeneous integration for on-chip quantum photonic circuits with single quantum dot devices. Nat. Commun..

[12] Zhang W, Yao J, Zhao YS (2016). Organic micro/nanoscale lasers. Acc. Chem. Res..

[13] Yang KY (2018). Bridging ultrahigh-Q devices and photonic circuits. Nat. Photon.

[14] Sirringhaus H, Tessler N, Friend RH (1998). Integrated optoelectronic devices based on conjugated polymers. Science.

[15] Ho PKH, Thomas DS, Friend RH, Tessler N (1999). All-polymer optoelectronic devices. Science.

[16] Hepp A (2003). Light-emitting field-effect transistor based on a tetracene thin film. Phys. Rev. Lett..

[17] Forrest SR, Thompson ME (2007). Introduction: organic electronics and optoelectronics. Chem. Rev..

[18] Hu W (2012). Organic Optoelectronics.

[19] Zhang X, Dong H, Hu W (2018). Organic semiconductor single crystals for electronics and photonics. Adv. Mater..

[20] Stratton, J. A. Electromagnetic Theory (Wiley-IEEE Press, New York, 2007).

[21] Melikyan A, Alloatti L, Muslija A (2014). High-speed plasmonic phase modulators. Nat. Photon.

[22] Reed GT, Mashanovich G, Gardes FY, Thomson DJ (2010). Silicon optical modulators. Nat. Photon.

[23] Jacobsen RS (2006). Strained silicon as a new electro-optic material. Nature.

[24] Yan R, Gargas D, Yang P (2009). Nanowire photonics. Nat. Photon.

[25] Greytak AB, Barrelet CJ, Li Y, Lieber CM (2005). Semiconductor nanowire laser and nanowire waveguide electro-optic modulators. Appl. Phys. Lett..

[26] Kastalsky A, Abeles JH, Leheny RF (1987). Novel optoelectronic single quantum well devices based on electron bleaching of exciton absorption. Appl. Phys. Lett..

[27] Dionne JA, Diest K, Sweatlock LA, Atwater HA (2009). PlasMOStor: a metal-oxide-Si field effect plasmonic modulator. Nano. Lett..

[28] High AA, Novitskaya EE, Butov LV, Hanson M, Gossard AC (2008). Control of exciton fluxes in an excitonic integrated circuit. Science.

[29] Abeles JH, Chat WK, Shokoohi FK, Bhat R, Koza MA (1987). Integration of GaAs MESFET drivers with GaAs directional-coupler electro-optic modulators. Electro Lett..

[30] Abeles JH, Chan WK, Colas E, Kastalsky A (1989). Junction field‐effect transistor single quantum well optical waveguide modulator employing the two‐dimensional Moss–Burstein effect. Appl. Phys. Lett..

[31] Wang C, Dong H, Hu W, Liu Y, Zhu D (2012). Semiconducting π-conjugated systems in field-effect transistors: a material odyssey of organic electronics. Chem. Rev..

[32] Zhao G (2012). Substitution effect on molecular packing and transistor performance of indolo[3,2-b]carbazole derivatives. J. Mater. Chem..

[33] Li R, Hu W, Liu Y, Zhu D (2010). Micro- and nanocrystals of organic semiconductors. Acc. Chem. Res..

[34] Lidzey DG (1998). Strong exciton–photon coupling in an organic semiconductor microcavity. Nature.

[35] Laudise RA, Kloc Ch, Simpkins PG, Siegrist T (1998). Physical vapor growth of organic semiconductors. J. Cryst. Growth.

[36] Snyder AW, Love JD (1983). Optical Waveguide Theory.

[37] Ye J (2014). Optical wavelength filters based on photonic confinement in semiconductor nanowire homojunctions. Adv. Mater..

[38] Liao Q, Fu H, Wang C, Yao J (2011). Cooperative assembly of binary molecular components into tubular structures for multiple photonic applications. Angew. Chem. Int. Ed..

[39] Zhang C, Yan Y, Zhao YS, Yao J (2014). From molecular design and materials construction to organic nanophotonic devices. Acc. Chem. Res..

[40] Yao W (2013). Controlling the structures and photonic properties of organic nanomaterials by molecular design. Angew. Chem. Int. Ed..

[41] Tang Q (2008). Micrometer- and nanometer-sized organic single-crystalline transistors. Adv. Mater..

[42] Tang Q (2007). Photoswitches and phototransistors from organic single-crystalline sub-micro/nanometer ribbons. Adv. Mater..

[43] Ji D (2013). Large scale, flexible organic transistor arrays and circuits based on polyimide materials. Org. Electron..

