# Author Correction: Organic field-effect optical waveguides

**DOI:** 10.1038/s41467-018-07909-0

**Published:** 2018-12-18

**Authors:** Guangyao Zhao, Huanli Dong, Qing Liao, Jun Jiang, Yi Luo, Hongbing Fu, Wenping Hu

**Affiliations:** 1 Beijing National Laboratory for Molecular Sciences, Key Laboratory of Organic Solids, Institute of Chemistry, Chinese Academy of Sciences, 100190 Beijing, China; 2 School of Chemistry and Chemical Engineering, University of the Chinese Academy of Sciences, 100039 Beijing, China; 3 Beijing Key Laboratory for Optical Materials and Photonic Devices, Department of Chemistry, Capital Normal University, 100037 Beijing, China; 4 Heifei National Laboratory for Physical Sciences at the Microscale, University of Science and Technology of China, 230026 Hefei, China; 5 Tianjin Key Laboratory of Molecular Optoelectronic Sciences, Department of Chemistry, School of Science, Tianjin University & Collaborative Innovation Center of Chemical Science and Engineering, 300072 Tianjin, China

Correction to: *Nature Communications*; 10.1038/s41467-018-07269-9, published online 15 November 2018

The original version of this Article contained an error in Fig. 3, in which the *x*-axes in Fig. 3e and Fig. 3f were incorrectly labelled ‘*V*_*ds*_’ rather than the correct ‘*V*_*gs*_’. This has been corrected in both the PDF and HTML versions of the Article.

