# Supplementary material for: Organic field-effect optical waveguides
Source: Nat Commun. 2018 Nov 15;9:4790. doi: 10.1038/s41467-018-07269-9 (PMC6237772; doi:10.1038/s41467-018-07269-9)
Supplement: Supplementary file 1 — Supplementary information [file 41467_2018_7269_MOESM1_ESM.docx]

*Supplementary Information for*

**Organic Field-effect Optical Waveguides**

Zhao et al.

**Supplementary Table of Contents**

**Supplementary Figure 1** | Principle of field-effect waveguides.

**Supplementary Figure 2** | Fluorescence property of CHICZ.

**Supplementary Figure 3** | Physical vapor transport system for CHICZ crystal growth.

**Supplementary Figure 4** | CHICZ single crystals grown by PVT method.

**Supplementary Figure 5** | AFM morphologies, height lines and photoluminescence images of CHICZ crystals.

**Supplementary Figure 6** | Size distribution of CHICZ single crystals fabricated by PVT method.

**Supplementary Figure 7** | X-ray diffraction patterns (XRD) of CHICZ crystals.

**Supplementary Figure 8** | Propagation loss of CHICZ single crystal ribbon.

**Supplementary Figure 9** | Device of individual CHICZ single crystal ribbon.

**Supplementary Figure 10** | OFET performance of individual CHICZ crystal.

**Supplementary Figure 11** | OFEW device based on individual CHICZ crystal.

**Supplementary Figure 12** | OFEWs measurement system.

**Supplementary Figure 13** | Optical waveguide property modulated by two-terminal devices.

**Supplementary Figure 14** | OFEWs measurement region to avoid optical cleaning.

**Supplementary Figure 15** | Theoretical calculations for modulation effect of gate voltage on the optical waveguide properties of CHICZ ribbons.

**Supplementary Notes**

**Supplementary Note 1:** **Principle of field-effect waveguides**

Photons could be imagined as a self-propagating transverse oscillating wave of electric and magnetic fields (Supplementary Fig. 1a). Hence, during the propagation of photons in optical waveguides, it has the potential to be tuned by external electric field. Simultaneously, taking field-effect transistor into consideration, charges transport in its conducting channel from source to drain forming the current, and such charge transport process will be also influenced by the incident optical waves according to electromagnetic wave theory (Supplementary Fig.1b).


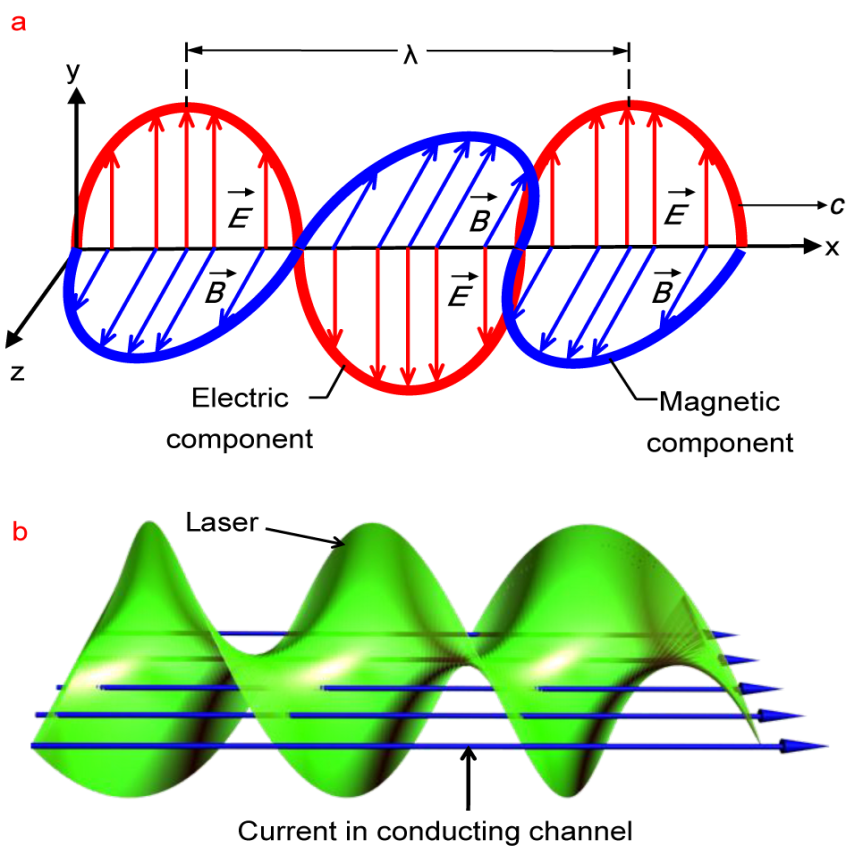


**Supplementary Figure 1 |** **Principle of field-effect waveguides**. **a**, Photons could be imagined as a self-propagating transverse oscillating wave of electric and magnetic fields. **b**, charge transport process will be tuned by the incident optical waves according to electromagnetic wave theory.

**Supplementary Note 2: Photophysical property of CHICZ crystals**

2,8-dichloro-5,11-dihexyl- indolo(3,2-b)carbazole (CHICZ) is a pentacene analogue, but different from pentacene without fluorescence, CHICZ shows strong fluorescence as shown in Supplementary Fig. 2a. Its crystal powders under UV light show strong fluorescence, indicating the good fluorescence property of CHICZ. UV-*vis* absorption and photoluminescence (PL) spectra of CHICZ crystals (Supplementary Fig. 2b) show a wide ultraviolet absorption peak among 375-475 nm, and two dominate narrowed PL peaks at 455 nm and 480 nm in PL spectrum, respectively. Moreover, CHICZ is very stable, which is due to its large energy gap of ~2.79 eV estimated from its long-wavelength absorption edge.

**
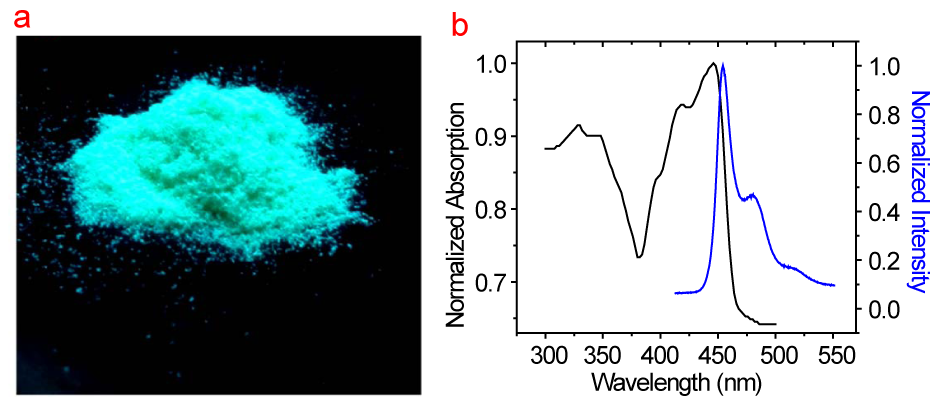
**

**Supplementary Figure 2 | Fluorescence property of CHICZ crystals. a,** The fluorescence image of CHICZ crystal powder under UV light. **b,** UV-*vis* absorption and photoluminescence (PL) spectra of CHICZ crystals.

**Supplementary Note 3:** **Physical vapor transport system for crystal growth**

The growth of CHICZ single crystals *via* physical vapor transfer (PVT) method is performed under atmosphere pressure with argon (Ar) as carrier gas as shown in Supplementary Fig. 3. PVT process is carried out by a tubular furnace which contains heating unit, quartz tube, temperature-controlled instrument, vacuum pump and so on. The PVT process includes the following steps: first, raw material evaporation at the sublimation region, second, CHICZ molecules carrying by Ar carrier gas, and third, recrystallization of CHICZ molecules at the crystallizing region. Here we heat raw material at a sublimation temperature at ~280 ^o^C, and CHICZ crystals are obtained after 10 h. CHICZ crystals usually stand on substrates and can be easily transferred for device fabrication.


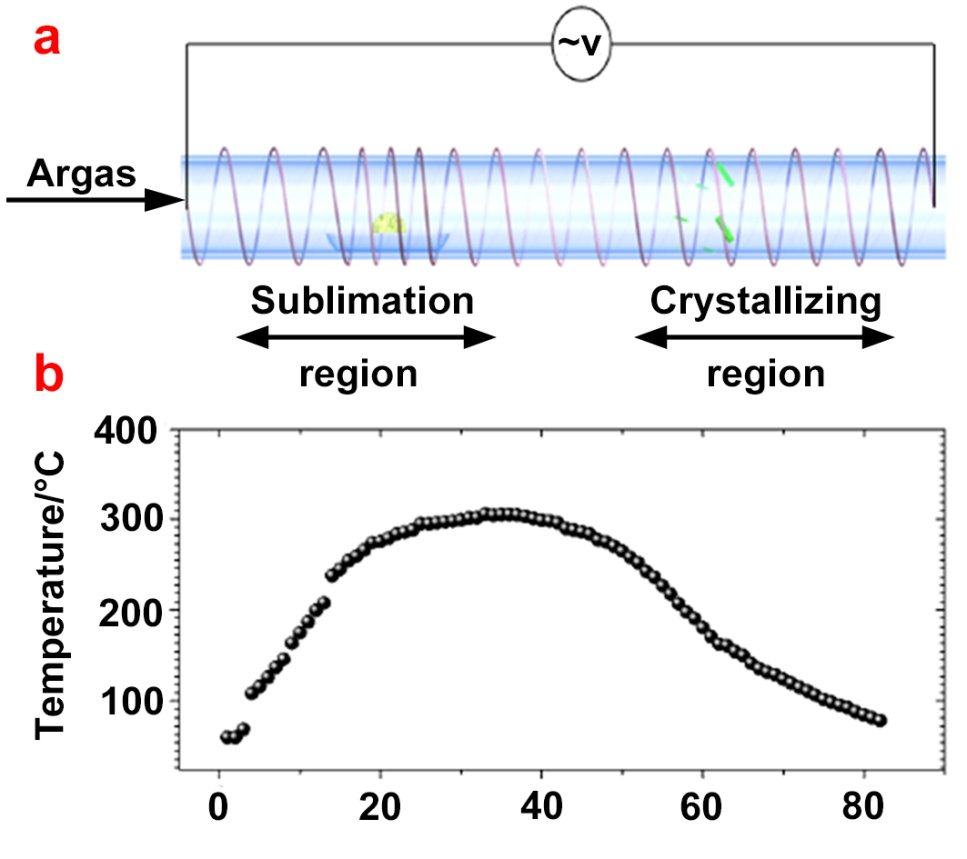


**Supplementary Figure 3 |** **Growth of for CHICZ single crystals**. **a,** PVT system for crystal growth. **b,** Temperature distribution in the furnace, which guides us to select proper sublimation region and recrystallization region.

Supplementary Note 4: CHICZ single crystals grown by physical vapor transport system (PVT) method

Supplementary Figs. 4-6 show the characterizations of CHICZ single crystals grown from PVT method. Supplementary Fig. 4a is a SEM image of CHICZ ribbon with length over 300 μm. Supplementary Fig. 4b is a photoluminescence micrograph of large size CHICZ sheet upon excitation with unfocused UV light (330–380 nm). It is obvious that CHICZ sheet shows large area, homogeneous photoluminescence, indicating the high quality of the sheet crystal. CHICZ crystals show high brightness at the edge areas and ends, indicating their excellent waveguide property.


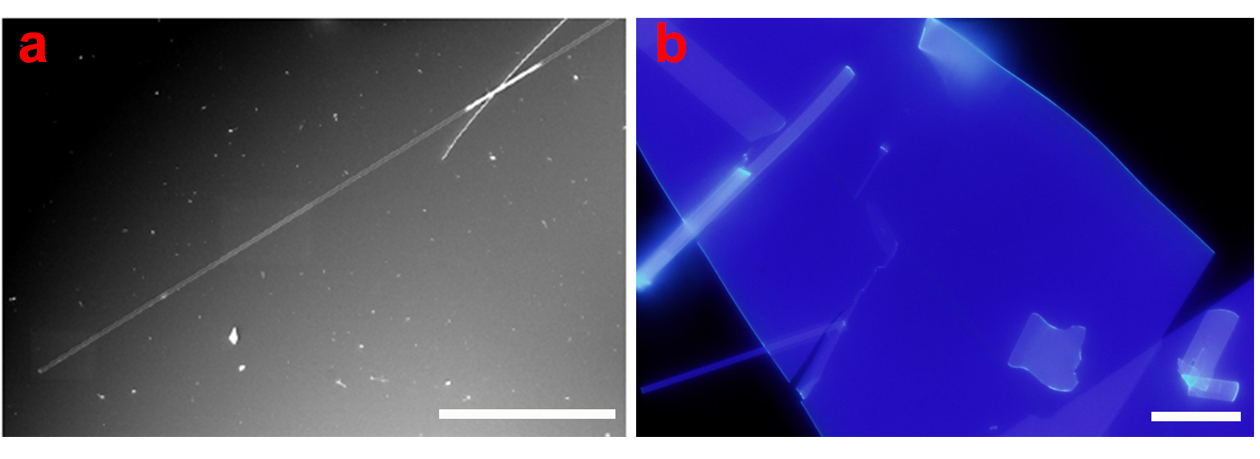


**Supplementary Figure 4 |** **CHICZ single crystals grown by PVT method**. **a**, Scanning electron microscopy (SEM) image of a CHICZ ribbon with length over 300 μm. Scale bar: 100 μm. **b**, Photoluminescence micrograph of a large size CHICZ sheet upon excitation with unfocused UV light (330–380 nm) illumination. Scale bar: 50 μm

Atomic force microscope (AFM) images of CHICZ ribbon crystals are shown in Supplementary Fig. 5. The heights of the crystals are hundreds nanometers. The smooth surface of the crystal indicates that the high quality of the ribbon crystal. As we know, high quality crystals are essential for the fabrication of organic field-effect transistors and organic optical waveguides, e.g., for high mobility and efficient photon transmission to reduce the electrical-optical loss to the maximum extent. Photoluminescence images show high brightness at the edge areas and ends, further confirming their potential excellent waveguide property.


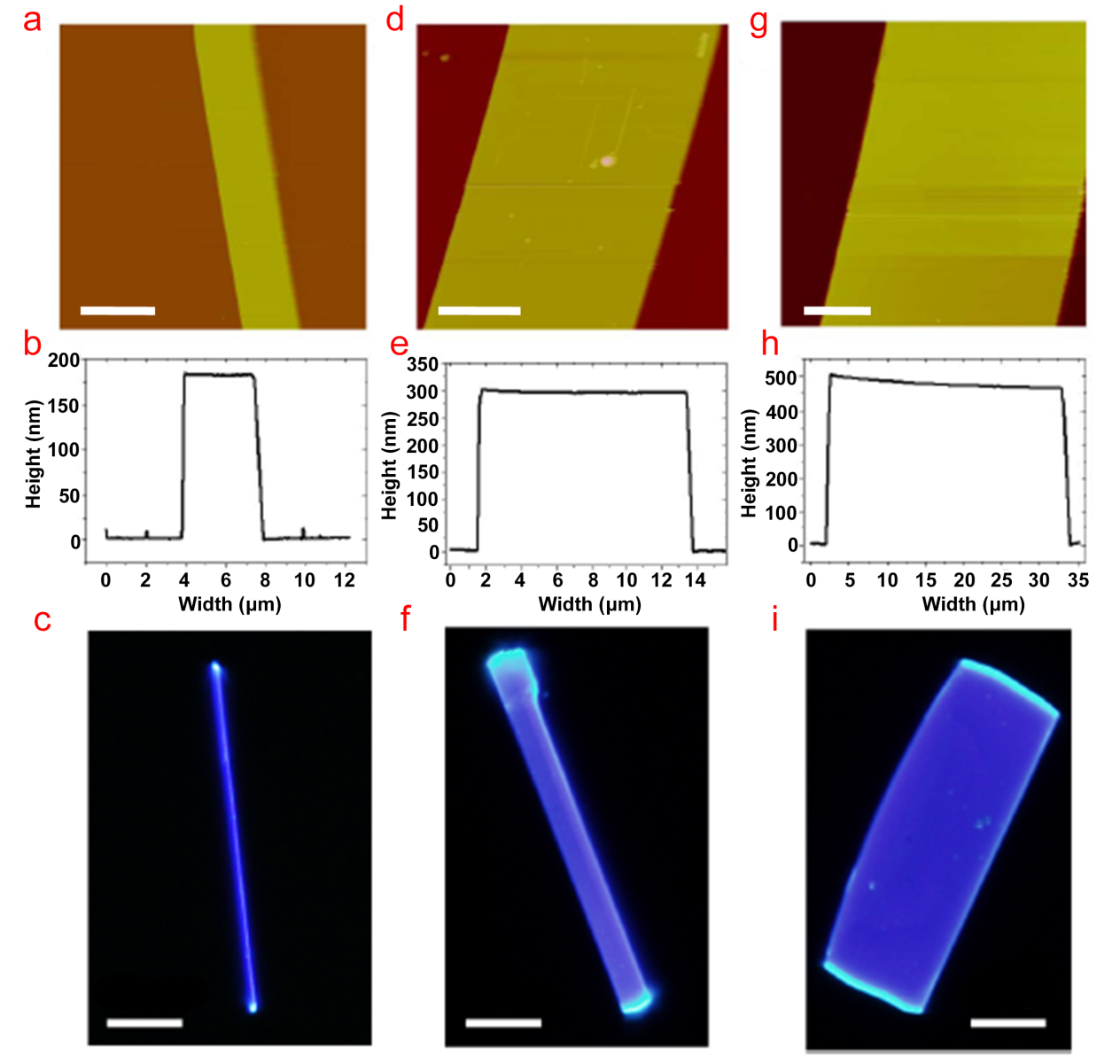


Supplementary Figure 5 | Characterizations of CHICZ single crystals. a,d,g, Atomic force microscopy (AFM) images of three different CHICZ crystals. b,e,h and c,f,i are their corresponding height lines and photoluminescence micrographs, demonstrating the high quality of the ribbon crystal. Scale bar: (a) 5 μm ; (b) 5 μm ;(c) 10 μm; (c,f,i) 20 μm.

The size distribution of CHICZ crystals obtained by PVT method is shown in Supplementary Fig. 6. The height of most crystals is ranged 100-500 nm, meeting the requirement of that with the sizes larger than that diffraction limit of light transmission of CHICZ material.

**
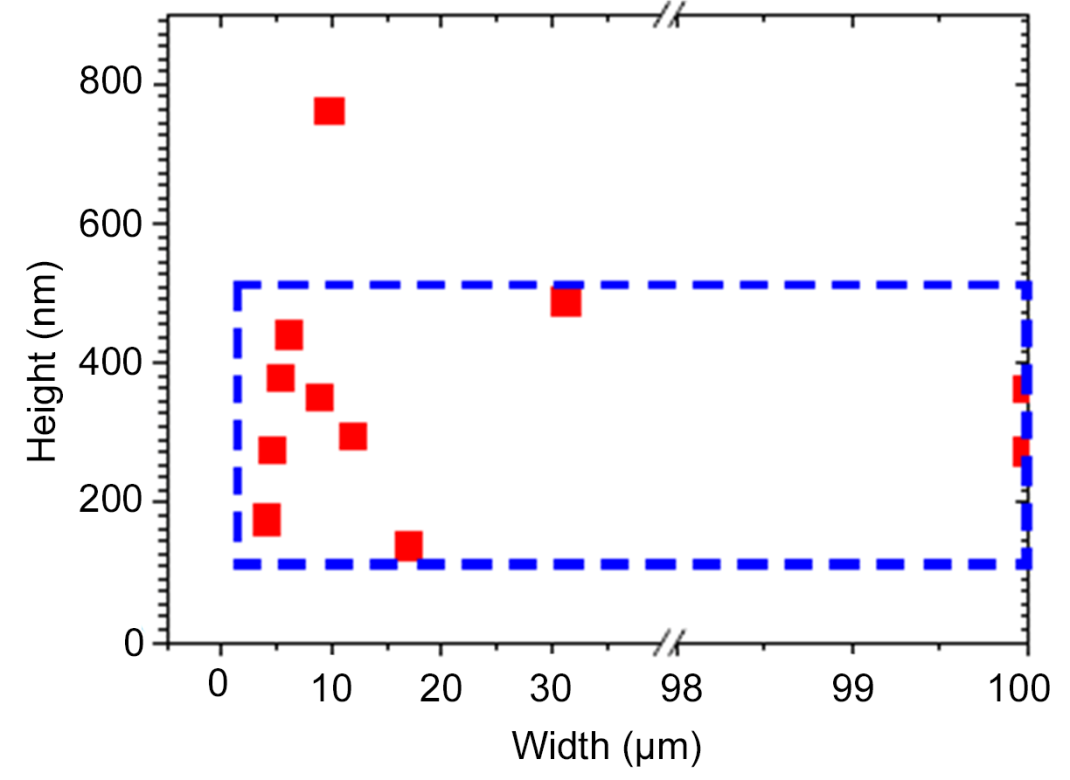
**

**Supplementary Figure 6 | Size distribution of CHICZ crystals.** Most of the single crystal with height ranged 100-500 nm.

Supplementary Note 5: X-ray diffraction patterns (XRD) of CHICZ crystals

X-ray diffraction (XRD) patterns of CHICZ crystals are shown in Supplementary Fig. 7. According to index of single crystal data, it is identified that the XRD patterns of CHICZ single crystals show only (h0l) (h=-l) patterns, indicating that the faces of CHICZ crystal are parallel to substrate and the [0 1 0] direction, i.e., the pi-pi stacking direction. This molecular packing motif is beneficial for charge transport.


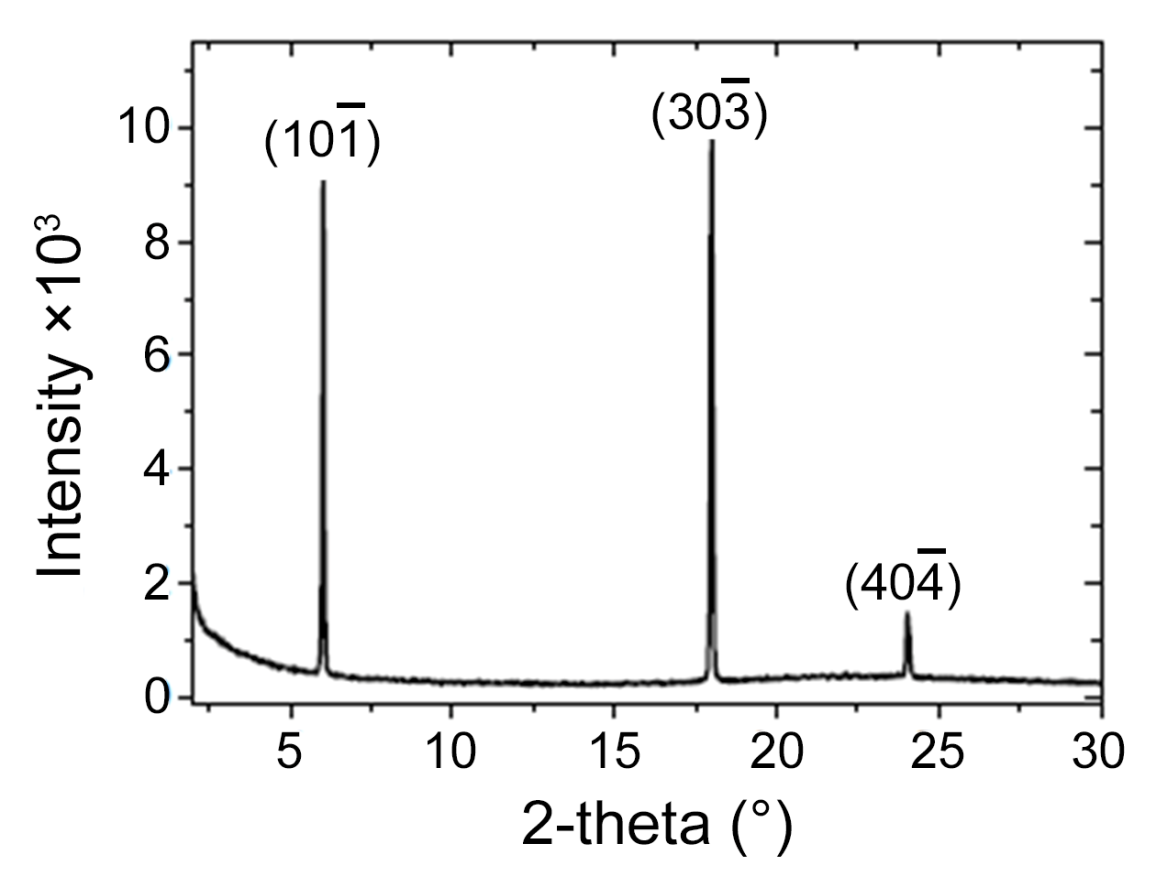


**Supplementary Figure 7 |** **X-ray diffraction of CHICZ crystals**. The patterns show only (h0l) (h=-l) patterns, indicating that the faces of CHICZ crystal are parallel to substrate and the [0 1 0] direction.

**Supplementary Note 6: Propagation loss of CHICZ single crystal ribbon**

The optical loss coefficient of the guided light in CHICZ single crystals is shown in Supplementary Figs. 8. Fig.8a shows loss coefficient measurements along the ribbon. With the laser spot moving from one end to another, the PL signal is changed with the travelled distance. The data of relative intensity *vs* length are showed in Supplementary Fig. 8b. The optical loss coefficient along the ribbon is calculated to be 10 dB/mm. Similarly, the optical loss coefficient crossed the ribbon is measured as shown in Supplementary Fig. 8c, and the value is calculated to be 20 dB/mm as shown in Supplementary Fig. 8d. All the results indicate the excellent waveguide property of CHICZ crystals.

**
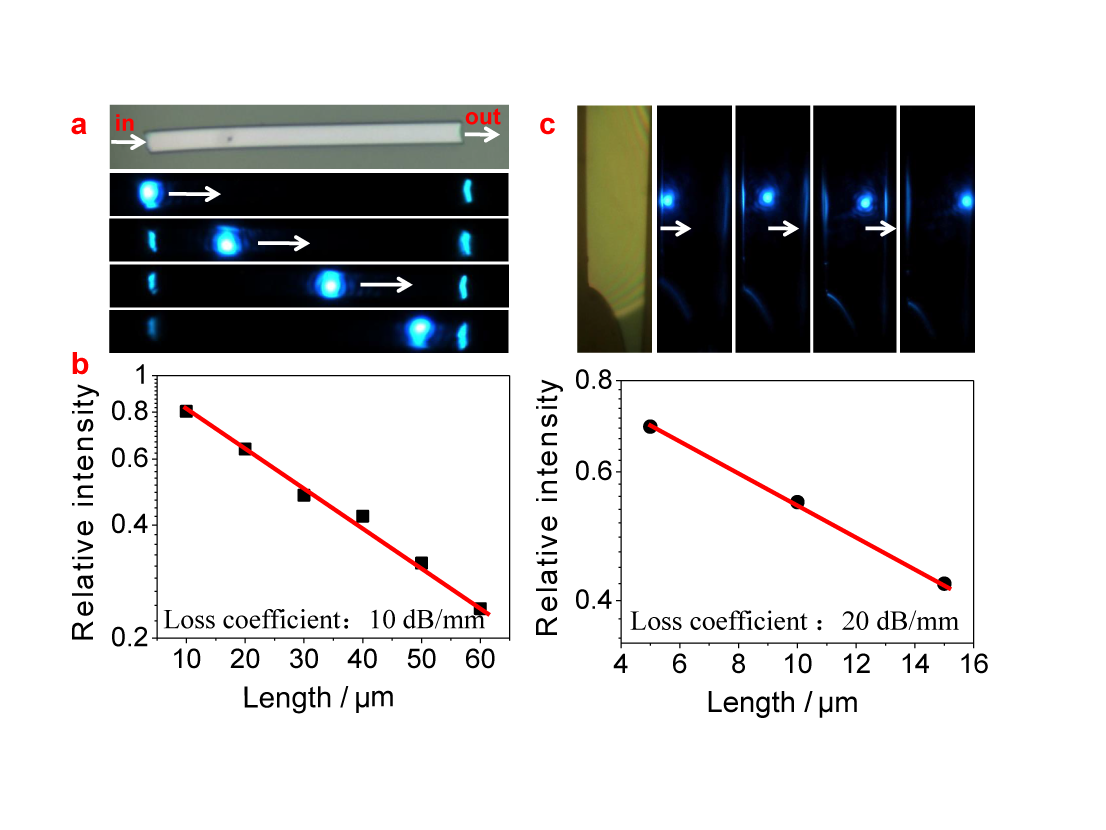
**

**Supplementary Figure 8 | Propagation loss of CHICZ single crystals**. **a**, Optical loss coefficient along the ribbon long axis. **b**, Dependence of relative PL intensity along the ribbon. **c**, Optical loss coefficient crossed the ribbon. **d**, Dependence of relative PL intensity crossed the ribbon.

**Supplementary Note 7: Device of individual CHICZ single crystal ribbon**

Top-contact, bottom-gate organic field-effect transistors (OFETs) of an individual CHICZ crystal are fabricated based on octadecyl-trichlorosilane (OTS) modified Si/SiO_2_ (300 nm) substrates using “gold-layer sticking technique” in order to avoid the damage of thermal radiation during the evaporation of source and drain electrodes. Here, a typical device is shown in Supplementary Fig. 9.


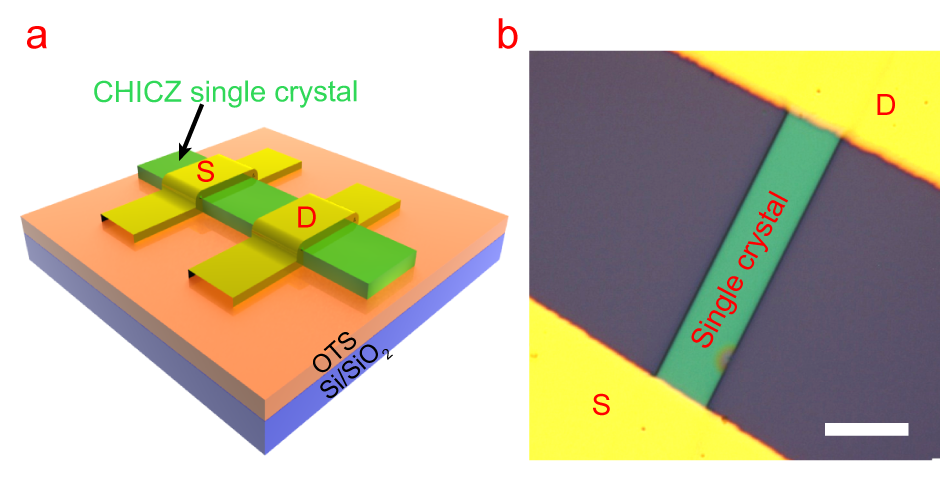


**Supplementary Figure 9 | OFET** **Device based on an individual CHICZ single crystal**. The devices are fabricated based on octadecyl-trichlorosilane (OTS)-modified Si/SiO_2_ (300 nm) substrates using “gold-layer sticking technique”. **a,** The schematic of organic field-effect transistor (OFET) based on an individual CHICZ single crystal. **b,** The optical microscopy image of a typical CHICZ-based OFET fabricated in the experiment. Scale bar: 10 μm.

**Supplementary Note 8: OFET performance of individual CHICZ crystal**

OFETs of individual CHICZ ribbon crystal exhibit excellent OFET property as shown in Supplementary Fig. 10. The output characteristics of the OFETs can be divided into linear and saturation regions. And from the saturated region or the transfer characteristics, the field-effect mobility and on/off ratio of the CHICZ single crystal are calculated to be 0.52 cm^2^V^-1^s^-1^ and 2×10^6^, respectively. Moreover, the corresponding threshold voltage of the device is around zero voltage. The high mobility, high on/off ratio, and low threshold voltage of field-effect transistors based on CHICZ crystals indicate their potential application in organic field-effect transistors.


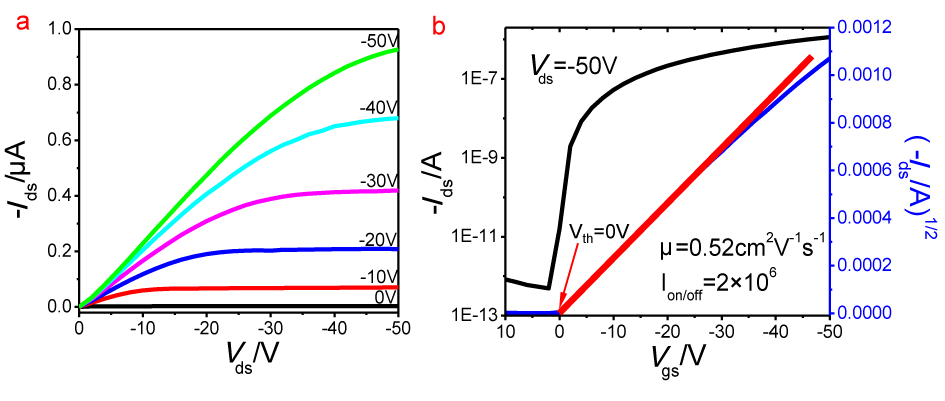


**Supplementary Figure 10 |** **OFET performance of CHICZ single crystal**. **a,** The output characteristics of the OFET, suggesting no obvious contact resistance existing in the large-size crystal devices. **b,** The transfer characteristics of the OFET, demonstrating ideal field-effect transporting property. The corresponding channel length and width are 36.2 and 7.2 μm, respectively.

**Supplementary Note 9: OFEW device based on individual CHICZ crystal**

Organic field-effect waveguides are fabricated based on individual CHICZ crystal and polyimide (PI)/indium tin oxide (ITO) substrate. PI film is spin-coated on ITO substrate with thickness of several hundred nanometers to 1 micrometer as gate insulator. Gold source and drain electrodes are vacuum evaporated on CHICZ ribbon crystal by “gold stripe mask” technique with thickness at around 40 nm. A typical final device is shown in Supplementary Fig. 11**.**


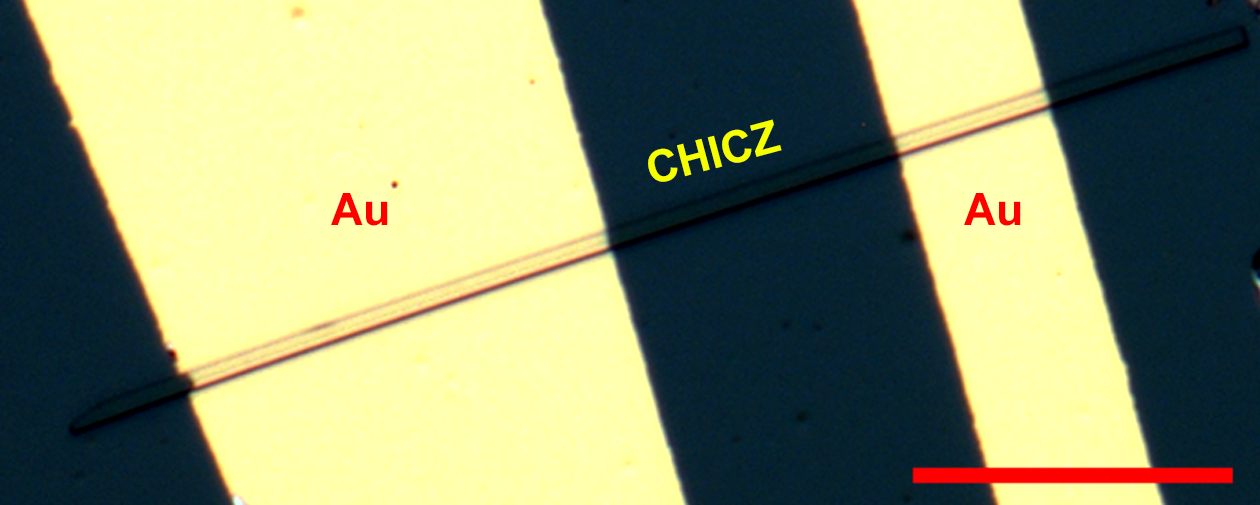


**Supplementary Figure 11 | Optical microscopy of a typical CHICZ-based OFEW**. Organic field-effect optical waveguides (OFEWs) are fabricated based on individual CHICZ crystal and polyimide (PI)/indium tin oxide (ITO) substrate with PI as insulator layer and gold film (40 nm) as source and drain electrodes. Scale bar: 50 μm

**Supplementary Note 10: OFEWs measurement system**

The measurement system includes the measurement systems for OFETs, optical waveguides, and laser etc. (Supplementary Fig. 12). All the measurements of organic field-effect waveguides (OFEWs) are carried out in ambient at room temperature.


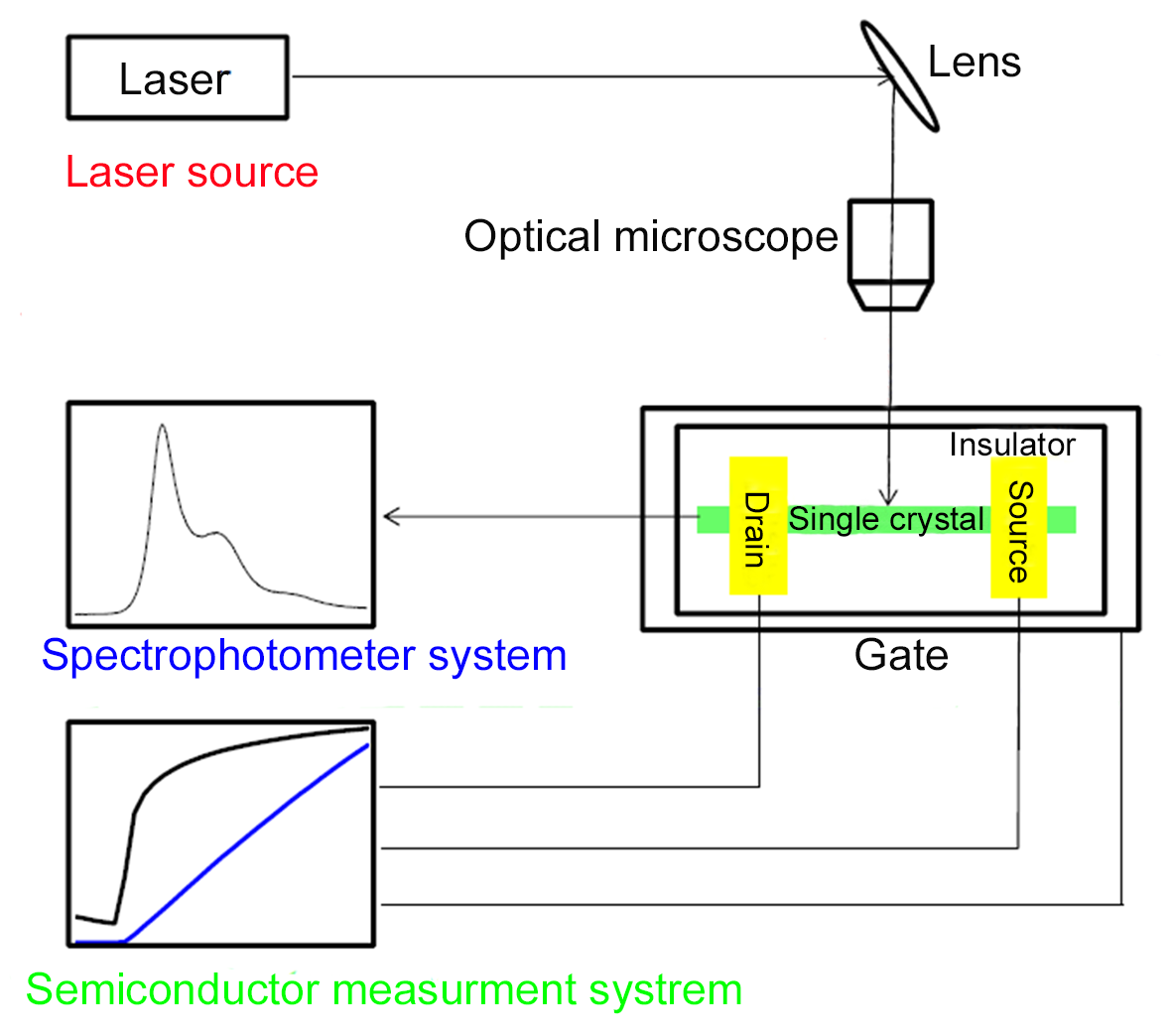


**Supplementary Figure 12 |** **OFEW measurement system**. The measurement system includes the measurement systems for OFETs, optical waveguides, and laser etc.

**Supplementary Note 11: Optical waveguide property modulated by two-terminal devices**

The lateral field induced between source-drain can also play a role on the modulation of optical waveguide performance for CHICZ single crystals. For a comparison, we had carried out the investigation without the gate based on a two-terminal device. The major difference is that without the gate the modulation depth is decreased with the modulation values of only 10% under the same source-drain voltage (as shown in Supplementary Fig. 13) probable due to the significant reduce of charge carrier density in the conducting channel without applying gate voltage. This result confirms the importance of signal amplification in three-terminal device architecture with great potential applications towards high-density and high-speed on-chip integrated optoelectronic circuits.

**
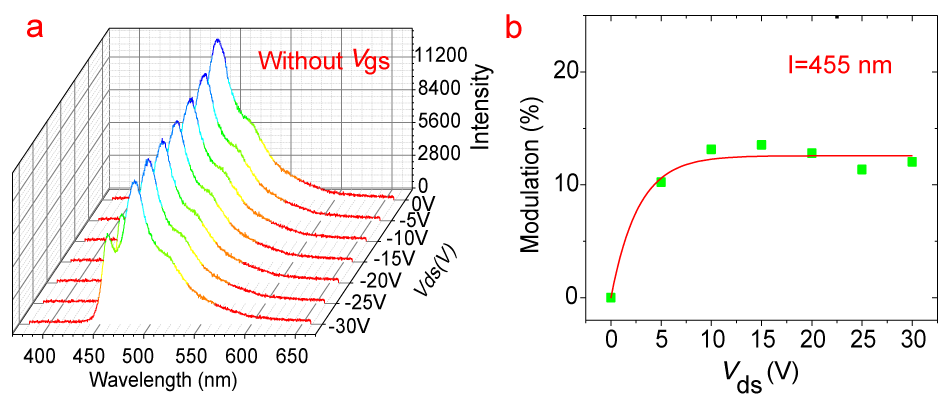
**

**Supplementary Figure 13 |** **CHICZ optical waveguide modulated by two-terminal device**. a) PL intensity dependence on source-drain voltage with current parallel to the optical waveguide. b) Modulation percentage of the waveguide intensity tuned only by source-drain voltage. The device channel length is 40 μm.

**Supplementary Note 12: OFEWs are measured to avoid optical cleaning**

For organic materials, optical cleaning is one of the most problems to be considered in field-effect waveguides operation. Long time optical cleaning measurement are carried out to investigate the optical cleaning property of CHICZ single crystals by recording their PL spectra every 2 minutes for 40 minutes. As shown in Supplementary Fig. 14, the PL intensities decrease linearly at the beginning 15 minutes which is called liner region, after then, the intensity remains stable in stable region. Because of this, the field-effect waveguide transistors are measured in stable region to eliminate the uncertain factors.

**
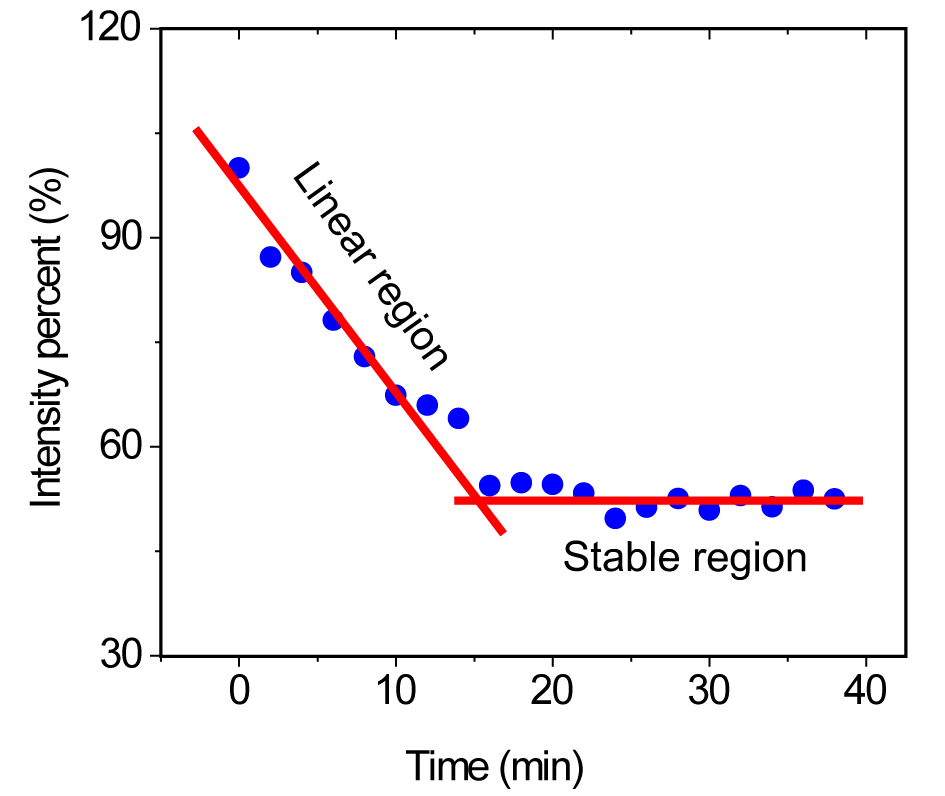
**

**Supplementary Figure 14 |** **Optical cleaning property of CHICZ crystals**. OFEWs are measured at the stable region to eliminate the uncertain factors.

**Supplementary Note 13: Theoretical calculations for modulation effect of gate voltage on the optical waveguide properties of CHICZ ribbons**

We have built atomic models and simulated electronic structures for the CHICZ species at the density function theory (DFT) level of using the Gaussian 09 program ^1^. Dimer models were built to examine the intermolecular coupling effects in our samples, in which two CHICZ are put in parallel pattern driven by weak interaction (π-π stacking) with a intermolecular distance of 3.5 Å (consistent with the experimental results in Fig. 1), as shown in Supplementary Figs. 15a,b. We used the hybrid functional B3LYP and 6-31G(d) basis to find the optimized geometries of monomer and dimer of CHICZ species. The highest occupied molecular orbital (HOMO) energies, the lowest unoccupied molecular orbital (LUMO) energies, and HOMO-LUMO gap were computed. In the neutral state, the HOMO-LUMO gap for dimers (3.6 eV) is similar with that of single molecule (3.7 eV), suggesting relatively weak intermolecular interaction between neighboring CHICZ molecules. The simulated optical excitation gap is about 2.95 eV after considering the optimized exited state and zero-point correction to thermal enthalpy, agreeing well with measured photo-absorption spectrum. It demonstrates that no matter for the single molecule and dimers, if a charge (positive or negative) was trapped by molecules when electric currents were driven by gate or source-drain voltage, the frontier orbitals would be effectively up- or down-shifted (Supplementary Figs. 15 b,e) which reduces the HOMO-LUMO gap significantly (i.e., from 3.7 eV to 2.0 eV and 1.6 eV for single molecule, and from 3.6 eV to 0.5 eV and 0.6 eV for dimers, respectively). The corresponding molecular orbitals in different states are shown in Supplementary Figs. 15 c,f. These break the energy match between adjacent molecules, and thereby shut down the channel for resonant-energy transferring. As a result, the waveguide will be heavily suppressed by the electron/hole trapping, through which we could achieve efficient voltage control of waveguide, as shown by the schematic image in Fig. 4 in manuscript.


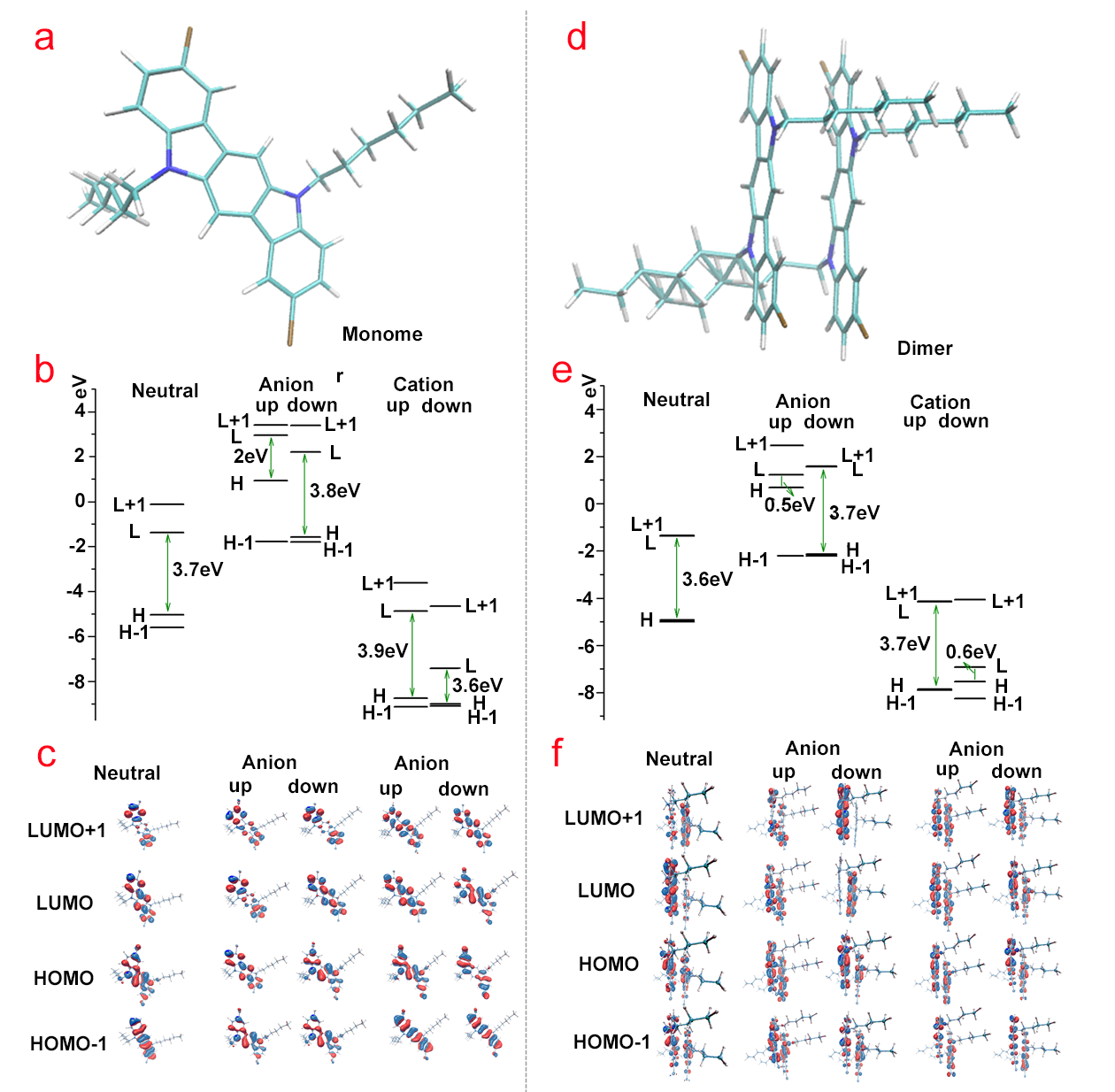


**Supplementary Figure 15 |** **The underlying mechanism of OFEWs**. The optimized molecular structure geometry for a) monomer molecule and its corresponding b) energy levels in neutral and charge trapped states as well as c) the molecular orbitals. The optimized molecular structure geometry for d) dimer built on the basis of the optimized monomer structure and its corresponding e) energy levels in neutral and charge trapped states as well as f) the molecular orbitals.

**Supplementary Reference**:

1. Frisch, M. J., Trucks, G. W. Schlegel, H. B. et al., Gaussian 09, Revision D.01, Gaussian, Inc., Wallingford, CT, (2009).
